# Human Variation in DNA Repair, Immune Function, and Cancer Risk

**DOI:** 10.3389/fimmu.2022.899574

**Published:** 2022-07-22

**Authors:** Ana Cheong, Zachary D. Nagel

**Affiliations:** Department of Environmental Health, Harvard T.H. Chan School of Public Health, Boston, MA, United States

**Keywords:** DNA repair, immunity, inter-individual variation, cancer risk, personalized medicine

## Abstract

DNA damage constantly threatens genome integrity, and DNA repair deficiency is associated with increased cancer risk. An intuitive and widely accepted explanation for this relationship is that unrepaired DNA damage leads to carcinogenesis due to the accumulation of mutations in somatic cells. But DNA repair also plays key roles in the function of immune cells, and immunodeficiency is an important risk factor for many cancers. Thus, it is possible that emerging links between inter-individual variation in DNA repair capacity and cancer risk are driven, at least in part, by variation in immune function, but this idea is underexplored. In this review we present an overview of the current understanding of the links between cancer risk and both inter-individual variation in DNA repair capacity and inter-individual variation in immune function. We discuss factors that play a role in both types of variability, including age, lifestyle, and environmental exposures. In conclusion, we propose a research paradigm that incorporates functional studies of both genome integrity and the immune system to predict cancer risk and lay the groundwork for personalized prevention.

## 1 Introduction

Why some individuals are more susceptible to cancer than others remains a fundamental unanswered question in cancer biology. Both immunodeficiency and DNA repair deficiency are associated with elevated cancer risk. The canonical hypothesis regarding DNA repair deficiency is that unrepaired DNA damage leads to increased somatic mutations and malignant transformation of somatic cells. An alternative, underexplored hypothesis is that DNA repair deficiency increases cancer risk, at least in part, by leading to impaired immune cell function. Immunodeficiency is associated with profound defects in some DNA repair pathways, but for some, like nucleotide excision repair, how they contribute to immune function is not yet understood. Furthermore, it remains unclear how inter-individual variation in immune function and DNA repair capacity (DRC) among the general population collectively contribute to cancer risk. We propose that integrating blood-based genome integrity assays and immunophenotyping could afford improved predictions of cancer risk and ultimately open new opportunities for precision prevention and treatment of cancer.

Here we provide an overview of the current understanding of the origins of inter-individual variation in both DNA repair and immune function, and the extent to which they have been shown to contribute to cancer risk. We have structured two sections on inter-individual variation in DNA repair (**Section 2**) and immune function (**Section 3**) similarly to underscore the many parallels between two fields that have largely developed independently. We discuss the role of each process in cancer risk, as well as genetic and non-genetic mechanisms contributing to inter-individual variation. After discussing the potential for integrating immunophenotyping and genome integrity assays into cancer risk prediction (**Section 4**), we highlight emerging technologies that are increasingly making such analyses feasible (**Section 5**), and close with a list of open questions recommendations for future studies (**Section 6**) and a brief synopsis (**Section 7**).

## 2 Variation in DNA Repair and Its Relationship to Cancer Risk and Carcinogenesis

### 2.1 DNA Repair Protects Against Cancer

**Figure 1:**
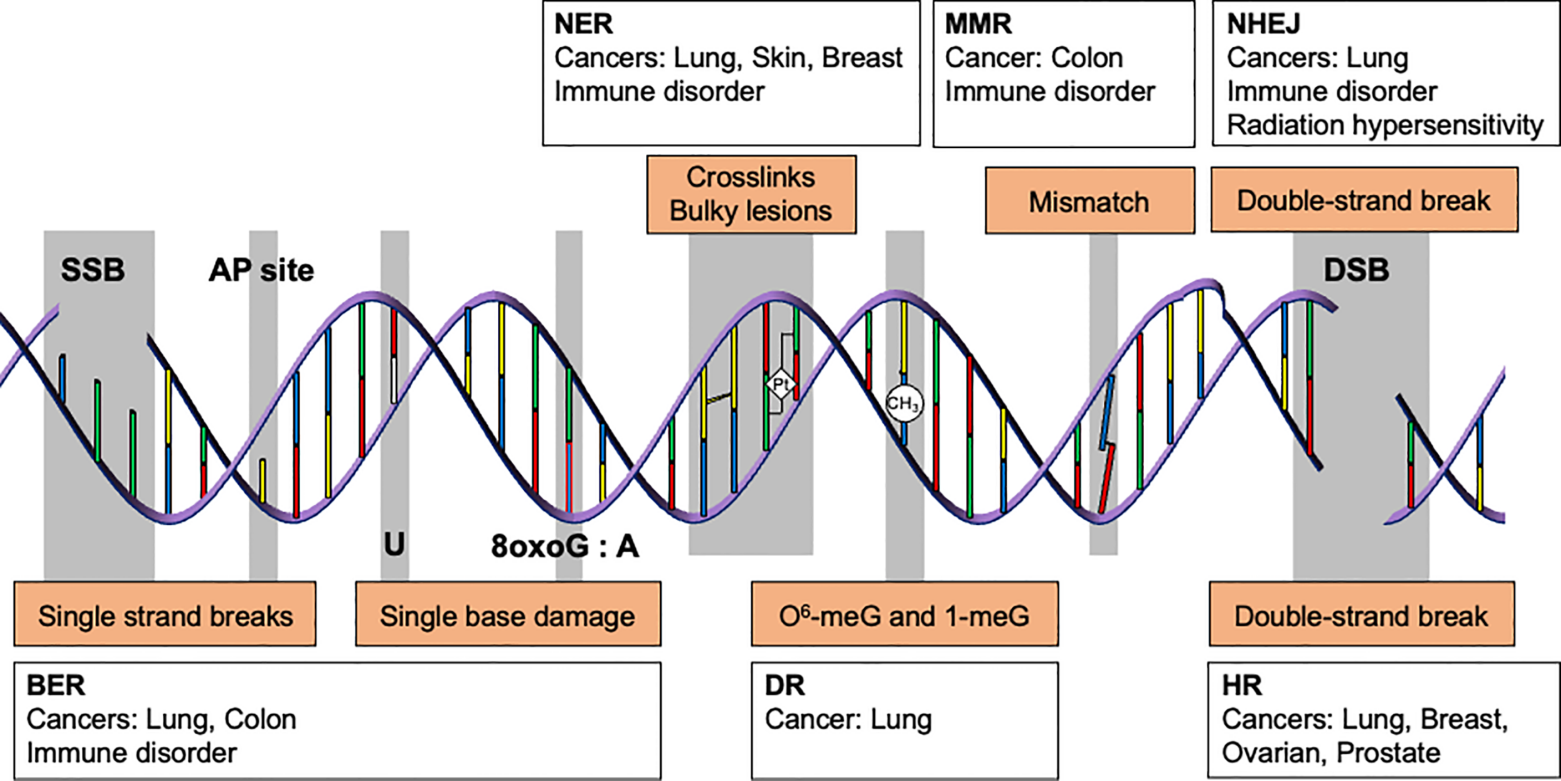
DNA repair pathways and their association with cancer and immune disorders. Genome integrity is maintained by multiple DNA repair pathways. Depending on the type of DNA damage, specific subsets of DNA repair proteins recognize and repair the damage. For instance, single strand breaks, abasic sites, and single base lesions are primarily repaired by base excision repair (BER). Some types of alkylation damage, such as *O* ^6^-methylguanine and 1-methylguanine, are repaired by direct reversal (DR). Intra-strand crosslinks and bulky lesions are repaired by nucleotide excision repair (NER). Mismatched bases are repaired by mismatch repair (MMR), whereas double strand breaks are resolved by homologous recombination (HR) or non-homologous end joining (NHEJ). Unrepaired DNA lesions may give rise to somatic mutations and cancer. Deficiency in BER, NER, MMR, and NHEJ is also associated with immunodeficiency, which increases cancer risk(s).

Genome integrity is constantly threatened by endogenous and environmental DNA damaging agents. These agents include reactive oxygen species (ROS) generated by normal cellular metabolism, errors in DNA replication, ultraviolet (UV) light, ionizing radiation, and mutagenic chemicals (1). While unrepaired DNA damage can lead to disease by promoting cell death, transcriptional stress, senescence, and mutations (2), DNA repair limits these processes by maintaining genome integrity. Depending on the agent, DNA can be damaged in numerous ways. The types of DNA damage include base damage, single strand breaks, inter- and intra-strand crosslinks, bulky adducts, methylated DNA adducts, mismatches, and double-strand DNA breaks (DSBs). Complexes of DNA repair proteins form DNA repair machines that specialize in the removal of particular types of DNA damage, and defects in one or more of the DNA repair pathways increase the frequency of specific types of mutations in the genome (3) (**Figure 1**). As DNA damage and repair have been extensively reviewed elsewhere (1, 4, 5), we will not cover the detailed mechanisms here.

### 2.2 Defects in DNA Repair Are Linked to Cancer-Prone Genetic Disorders

Genome instability is a hallmark of cancer, and nearly all cancers are caused by one or more somatic mutations induced by DNA replication in the presence of DNA damage (6, 7). As our understanding of the etiology of cancer mutation signatures advances rapidly, it is becoming evident that genomic alterations in individual cancers can often be attributed to specific DNA damaging agents and DNA repair defects (3, 8–11). Historically, much has been learned from constitutional DNA repair deficiency syndromes that are associated with elevated cancer risk in humans. Below we highlight examples for several DNA repair pathways. In subsequent sections, we discuss variability in DNA repair in the general population, which is emerging as a potential predictor for cancer risk (12, 13) (14) (15).

Nucleotide excision repair defects in xeroderma pigmentosum (XP) patients are associated with an extremely high risk of skin cancers due to the inability to repair UV-induced DNA damage (16). Early studies revealed seven complementation groups that correspond to the DNA repair genes XPA, XPB, XPC, XPD, XPE, XPF, and XPG. Deficiency in the translesion DNA polymerase eta (aka POLH or XPV) also causes XP in humans (17) (18). Cells from these individuals exhibit normal NER, but are deficient for accurate replicative bypass of unrepaired UV-induced DNA damage, resulting in an increased rate of UV-induced mutagenesis. In the case of combined XP and Cockayne syndrome (XP-CS), mutations in *XPB*, *XPF*, *XPD*, or *XPG* have been detected among patients. These patients display a mild XP phenotype. Yet, despite the universality of DNA repair deficiency, skin cancers are rare except in those with mutations in *XPB* or *XPD* (19). XP-CS patients with mutations in XPG are photosensitive and have skin freckling, but skin cancers are rare. This may be in part due to very early mortality, but the severe photosensitivity phenotype that commonly accompanies XPG-CS also leads to early diagnosis and better sun protection for these patients.

Numerous diseases are associated with defects in double strand break repair. Fanconi Anemia is caused by mutations in a group of genes involved in both DSB repair and the repair of DNA inter-strand cross-links (20). Patients commonly experience immunodeficiency due to bone marrow failure and are at increased risk of acute myeloid leukemia (21). Mutation in Werner syndrome protein (WRN) predisposes to cancer. WRN is a RecQ family DNA helicase with well-established roles in both non-homologous end joining (NHEJ) and homologous recombination (HR), as well as emerging roles in base excision repair (BER) and nucleotide excision repair (NER) (22). Patients with Werner syndrome display premature aging, and have higher risks of cancer and cardiovascular disease (23–26). WRN patients develop thyroid epithelial neoplasms, melanoma, and soft tissue sarcomas, as well as leukemia and primary bone neoplasms (27). RECQL4 is involved in NHEJ (28, 29), HR (30), NER, and BER (31), and its mutation is known to induce trisomy, aneuploidy, and chromosomal rearrangements. RECQL4 deficiency is associated with several diseases, including Rothmund-Thomson syndrome (RTS), RAPADILINO syndrome, and Baller-Gerold syndrome (BGS) (32). Patients with RTS or RAPADILINO have higher risk for osteosarcoma and lymphoma (33, 34). LIG4 syndrome is caused by deficiency in Ligase IV, which is essential for NHEJ (35). Patients with LIG4 syndrome exhibit severe combined immunodeficiency due to the role of NHEJ in V(D)J recombination, a key process for antibody diversification (36). Ataxia telangiectasia (A-T) is a DNA damage response disorder caused by mutations in the Ataxia telangiectasia mutated (ATM) gene. Among other symptoms, patients with A-T experience immunodeficiency and are at increased risk for cancer, particularly lymphoid malignancies (37).

Several diseases are associated with defects in base excision repair and single strand break repair (38) (39). MutY DNA glycosylase (MUTYH) -associated polyposis (MAP) arises from germline mutation of MUTYH. Characterized mainly by the biallelic germline mutations of Y165C or G382D in MUTYH, MAP is associated with colorectal adenomas and carcinomas (40, 41). As a BER protein, MUTYH functions to remove adenine opposite 8-oxo-7,8-dihydroguanine (OG), which is left unrepaired by 8-oxoguanine DNA glycosylase (OGG1), and thereby prevent G:C to T:A transversion mutations (42). Some MUTYH variants are associated with diminished OG:A repair (43), leading to higher colorectal cancer (CRC) risk (44) (45). Defects in uracil DNA glycosylase (UNG) result in an extreme immunodeficiency known as Hyper-IgM syndrome due to the central role of this enzyme in antibody diversification (46). While UNG deficiency is too rare to allow reliable estimates of its consequences for cancer risk, in general Hyper-IgM patients suffer from higher rates of malignancy (47). Similarly, deficiency in the Nth like DNA glycosylase 1 (NTHL1) is associated with a tumor syndrome that is dominated by colorectal cancer but includes several other malignancies (48–50).

Constitutional mismatch repair deficiency is an extremely rare disease that is associated with increased risk of a wide range of malignancies (51). Lynch syndrome is another DNA repair deficiency syndrome associated with cancer. It arises from the presence of one or more mismatch repair (MMR) gene mutations (52). While the normal tissues in Lynch syndrome patients often do not exhibit detectable MMR defects, Lynch syndrome is associated with MMR-deficient cancers with high microsatellite instability (MSI) (53).

### 2.3 Factors That Contribute to Variation in DRC

While much has been learned from diseases associated with DNA repair deficiency, they are relatively rare and represent the extremes of inefficient DNA repair in human populations. In the general population, DNA repair gene polymorphisms, age, environmental exposures, and lifestyle are several major factors thought to give rise to inter-individual variation (12). Variation in DRC is a consequence of the collective influence of these factors.

#### 2.3.1 Genetics

A large number of polymorphisms have been identified in DNA repair genes, and their associations with cancer imply functional consequences. While relatively few studies have investigated functional significance directly, accumulating research supports genetic variation as an important driver of inter-individual variation in DRC (**Table 1**). For example, variant alleles of *X-Ray repair cross complementing 3* (*XRCC3*) are associated with higher levels of bulky DNA adducts (59). *XRCC1* variants may be associated with either higher or lower BER repair activities (60, 62–64). *XPD* polymorphisms decrease XPD expression, with the most pronounced effect seen in older individuals (55). Some *OGG1* variants are associated with higher percentage tail DNA measured using comet assays (% tail DNA). Variant genotypes of BER and NER genes have also been associated with a wide variety of markers of genome instability. These include micronuclei and baseline %TD (58, 61, 66), chromosome breaks (62), sister chromatid exchanges (56, 60, 61), deletions and dicentric chromosomes (56), DNA adduct levels (59), overall BER repair activities (65), repair of radiation-induced damage (54) (58) (56), and repair of oxidative damage (54) (43), with cumulative effects for individuals with variant alleles in multiple DNA repair genes (57). While genetic determinants of DRC might be presumed exert similar effects on all tissues, this may not be true in light of evidence from animal models indicating tissue-dependent allele specific expression (67).

**Table 1:** Polymorphism in DNA repair genes and their association with genome integrity.

Genes	Genotype	DNA damage and repair activities	Ref.
Base excision repair			
*OGG1*	Ser326Cys; GG	Lower OGG1 activity vs. CC and CG genotypes	(54)
* *	Ser326Cys	Higher DNA damage vs OGG1 326 Ser/Ser genotype	(55)
* *		Inefficient repair of oxidative DNA damage ^a^	(54)
*MUTYH*	G382D, Y165C, and Q324H	Less efficient in repairing 8oxoG:A mispairs vs. wild-type MUTYH	(43)
*APE1*	Asn148Gln	Inefficient repair of oxidative DNA damage	(54)
* *		Associated with repairing of X-ray induced DNA damage	(54, 56)
* *		Associated with mitotic delay following X-irradiation	(57)
Nucleotide excision repair			
*ERCC/XPC*	Lys939Gln	Associated with repairing of X-ray induced DNA damage	(58)
*ERCC2/XPD*	D312N in exon 10	reduced XPD expression ^b^	(55)
* *	K751Q in exon 23	reduced XPD expression ^b^	(55)
* *	R156R in exon 6	reduced XPD expression ^b^	(55)
* *	312Asn	Not associated with repair of X-ray induced DNA damage	(56)
* *		Reduction in dicentric chromosomes and two-fold increase in translocation and chromatid exchange	(56)
* *	751Gln	Not associated with repair of X-ray induced DNA damage	(56)
* *		Reduction in dicentric chromosomes and two-fold increase in translocation and chromatid exchange	(56)
* *	Lys751Gln	Higher levels of bulky DNA adducts	(59)
* *		Not associated with higher mean SCE frequency ^c^	(60)
* *	Gln751Gln	Higher SCE frequency vs. Lys/Lys and Lys/Gln	(61)
Single strand break repair			
*XRCC1*	399Gln	Lower BER activities	(60, 62–65)
* *		Associated with repair of X-ray induced DNA damage	(56, 58)
* *		Higher mean SCE frequency ^c^	(60)
* *		Increase in deletions	(56)
* *	Arg399Gln	Lower irradiation-specific DNA repair rates	(54)
* *		Associated with mitotic delay ^d^	(57)
* *	Arg399Gln; Gln/Gln	More chromosome breaks per cell vs. other genotypes	(62)
* *	Arg399Gln; AA	Higher DNA adduct levels vs. AG and GG genotypes among non-smokers	(59)
	194Trp	Higher BER activities	(60, 62–64)
	194Try	Not associated with repair of X-ray induced DNA damage	(56)
* *		Increase in chromatid exchange	(56)
* *	Arg194Try	Inefficient repair of oxidative DNA damage	(54)
* *	Arg194Try; Arg/Arg	More chromosome breaks per cell vs. other genotypes	(62)
* *	Arg280His	Inefficient repair of oxidative DNA damage	(54)
Double strand break repair			
*XRCC3*	Thr241Met	Higher levels of bulky DNA adducts	(59)
* *	241Met	Not associated with repair of X-ray induced DNA damage	(56)
* *		Increase in deletions	(56)

a dominant effect, with repair capacity of oxidative DNA damage decreases with increasing number of variant alleles in OGG1 Ser326Cys and in combination with other gene polymorphisms (XRCC1 Arg194Try, Arg280His, and Arg399Gln, and APE1 Asn148Glu).

b either single or in combination, reduced XPD expression.

c independent of age, race, and family history of lung cancer.

d only among individuals with family history of breast cancer.

#### 2.3.2 Aging

An age-dependent decline in DRC and DNA damage accumulation has been proposed as a key mechanism underlying aging (68), and ongoing studies are beginning to uncover interventions that may mitigate the effects of compromised genome integrity in older individuals (69). The presence of age-dependent changes and the potential for interventions that may reverse them underline the likely role for age in inter-individual variation in DRC. Here we highlight studies testing this idea directly in human populations.

Assays that measure the accumulation of DNA damage provide indirect evidence for age-dependent changes in DRC. Peripheral blood mononuclear cells (PBMCs) isolated from older individuals have a higher frequency of dicentric and ring chromosomes (70) and a higher degree of negative supercoiling (71). Levels of single-strand breaks (SSBs) and oxidized bases (detected as formamidopyrimidine DNA glycosylase (FPG)-sensitive sites) in PBMCs are lower in younger individuals (age <65 years) when compared with older individuals (age >65 years) (72), although basal levels of SSBs and alkali sensitive sites in lymphocytes were age-independent in a separate study (73).

Direct measurements of DNA repair provide further insights into age-dependent changes in genome integrity. A study that used neutral comet assays to measure double strand break (DSB) repair and fluorometric analysis of DNA unwinding (FADU) to measure SSB repair in X-irradiated lymphocytes found diminished DSB repair in older individuals (74). Another study found that while overall rates of repair were similar, a subpopulation of repair deficient lymphocytes was significantly more abundant in older individuals (73, 74). Higher levels of DNA damage might intuitively be interpreted to reflect inefficient DNA repair, but the situation may be more complex. For example, one study found that the level of SSBs correlated positively with OGG1 activity (72), which was higher in older individuals. The higher levels of SSBs may thus reflect the accumulation of unresolved repair intermediates downstream of BER initiation, and phenomenon that has been termed BER imbalance (75–79). Elevated OGG1 activity in lymphocytes from older individuals has been observed in additional studies (72, 80). Nevertheless, in another study OGG1 repair activity in lymphocytes was reported to decrease with age (81). The decrease in OGG1 activity was more pronounced among individuals with Cys/Cys, Ser/Cys, than with Ser/Ser genotypes at position 326, suggesting that study design and the genetic makeup of cohorts may at least partially explain the differences among studies. By contrast with OGG1, AP site incision capacity is not associated with age (82).

Evidence for age-dependent changes in DRC have also come from studies wherein cells have been challenged with DNA damaging agents. Repair replication declines in lymphocytes irradiated with ultra-violet light (UV), with the rate of UV-induced decrease in DRC estimated to be about 30% from 20 to 90 years (83). By contrast, repair replication in UV-irradiated keratinocytes is comparable between infant and older adults, suggesting that age effects may be heterogeneous across human tissues (84). Similar to the decline in repair of UV-induced damage, rejoining of chromosomes following X-irradiation decreases with age in human leukocytes (85). Consistent with higher rates of BER initiation following oxidative DNA damage, a study that compared individuals in three groups based on age of 35-39 years (Group 1), 50-54 years (Group 2), and 65-69 years (Group 3) using an ELISA assay in PBMCs following challenge with hydrogen peroxide revealed significantly higher levels of single stranded DNA in Group 3, but not Group 2, when compared to Group 1 (86). This finding is consistent with a second study that made use of comet assays (80),, as well as those in the previous section finding elevated OGG1 activity in older individuals. A rare *in vivo* study in which the epidermis of subjects was subjected to UV-irradiation followed by skin biopsies found that the efficiency in removing irradiation-induced cyclobutane pyrimidine dimers (CPD) is lower in older subjects, consistent with *ex vivo* studies in cultured primary cells (87).

Host cell reactivation assays have provided important insights into age-dependent changes in DRC. For instance, in one study skin fibroblasts from younger donors had higher efficiency in repairing UV-irradiated plasmids than those from the older donors (88). However, the same study found no relationship between age and the removal of genomic UV-induced adducts, and a second study found the repair UV-induced induced plasmid lesions was similar in skin fibroblasts from donors of age 21-96 years (89). The differences between the HCR studies in fibroblasts might reflect the relatively small samples sizes (N=8-10), which limit statistical power; a somewhat larger study (N=20) using host cell reactivation assays in fibroblasts did find an age-dependent decrease in DRC (90). In lymphocytes, repair of UV irradiated plasmids decreases with age, with an estimated average 0.61% decrease per year between 20 and 60 years of age (91). These results were consistent with a second study using HCR in lymphocytes that found an age-dependent decline in repair of UV-induced damage, which was notably absent among basal cell carcinoma cases, for whom DRC was lower than in controls at younger age (92). Another study that stands out for its analysis of pathways other than NER using host cell reactivation assays in primary skin fibroblasts indicated that both NHEJ and HR decline strikingly with age (93). Taken together, the findings suggest that age-dependent changes in DRC may depend in a complex manner on the cell type, DNA repair pathway, and the health status of the study participants.

Age-associated changes in DRC may be explained in part by the differential expression of DNA repair genes. The expression levels of excision repair cross-complementing group 1 (ERCC1) (94, 95), XPA (94), XPF (95), XRCC4, ligase 4 (LIG4), LIG3 (93), DNA polymerase delta 1 (POLD1) (88, 96), POLE, replication factor C (RFC) (88), and replication protein A (RPA) (94) decrease with age. On the contrary, the expression levels of CSA and XPG seemed to increase with age, but the change could not be confirmed by qPCR (88). There is no difference in the expression levels of proliferating cell nuclear antigen (PCNA) (88), NHEJ factors DNA PKcs, artemis, XRCC4-like factor (XLF) (93),, Ku70 and Ku80 (93, 97) and HR factors breast cancer associated gene 2 (BRCA2), meiotic recombination 11 (MRE11), RAD51, Nijmegen breakage syndrome 1 (NBS1), and RAD51 (93) among different age groups. While these studies were performed in either human PBMCs and primary fibroblasts, whether these changes in the expression of DNA repair factors resemble those in other tissues from the same individual have not been studied.

Though a detailed review of animal models is beyond the scope of this article, we note that they recapitulate several aspects of human aging biology with respect to genome integrity, including age-dependent increases in DNA damage levels (98) (99), accumulation of mutations (100, 101), and dysregulation of DNA repair (102) (103) (104) (105) (106).

#### 2.3.3 Environmental Factors

Mounting evidence indicates that environmental exposures can alter DRC. Here we focus on two well-established examples, namely arsenic and smoking. Like tobacco smoke, arsenic is an environmental agent classified as carcinogenic to humans by the International Agency for Research on Cancer (107), and causes cancer at least in part by directly inducing DNA damage (108, 109). Exposure to arsenic is associated with chromosome aberrations in human PBMCs (110) and DNA damage (111, 112). Children with *in utero* exposure to arsenic have higher salivary 8-hydroxydeoxyguanosine (8-OHdG), a biomarker of DNA damage caused by oxidative stress, than their unexposed counterparts (113) (114). Consistent with a key role for DNA damage in the etiology of arsenic associated malignancies, arsenic exposure is associated with a distinct mutational signature (115). Furthermore, individuals with lower DRC and those with select polymorphisms in DNA repair genes are more susceptible to arsenic induced skin lesions (109) (108) (116, 117) (110) (118).

Population studies provide extensive evidence in support of the concept that arsenic exposure leads to alterations in DNA repair. Arsenic exposure is associated with decreased expression of *MutS homolog 2* (*MSH2*) and mutL homolog 1 (*MLH1*), though not PMS1 homolog 2 (*PMS2*) (119). Urinary arsenic concentrations are positively associated with *MLH1* promoter methylation, which is consistent with an epigenetic mechanism of arsenic-induced dysregulation of MMR (120). Arsenic exposure also leads overexpression of excision repair cross complementation group 2 (ERCC2/XPD) and less efficient NER (121). *ERCC1* expression may be influenced by arsenic exposure (122) (111), but there appear to be complex dependencies on dose, duration, and speciation of arsenic exposure (122), as well as the age of the exposed population (94, 95). Diminished expression of *XPF* and *XPB*, but increased in *XPG* expression have been associated with arsenic exposure (123). At the functional level, repair of DNA damage induced by hydrogen peroxide, ionizing radiation and 2-acetylaminofluorene (2-AAF) is impaired in arsenic-exposed individuals relative to unexposed controls (112) (117) (111). These population studies are broadly consistent with *in vitro* studies indicating that arsenic exposure synergizes with the DNA damaging effect of UV (124, 125) and inhibits repair of DNA damage induced by a variety of agents (124) (126) (127). Collectively, these findings indicate that, in addition to the direct induction of DNA damage, arsenic exposure likely sensitizes cells to the DNA damaging effects of other mutagenic agents.

Exposure to environmental tobacco smoke (ETS), also known as passive smoking, compromises genomic stability. Passive smokers have higher levels of several types of DNA damage than unexposed individuals (128). They also excrete higher levels of 5-hydroxymethyluracil (129), which is not directly induced by tobacco smoke but may result from ETS-induced oxidative stress. Though passive smoking has not been correlated with levels of 8-OHdG in serum or leukocyte DNA (128, 130), lymphocytes from passive smokers have a longer comet tail length, more Fpg-sensitive sites, and are slower in repairing H_2_O_2_-induced DNA damage (131). Furthermore, buccal epithelial cells of passive smokers have higher micronuclei frequency when compared to non-smokers (132). Interestingly, allele variants and expression levels of several DNA repair genes have been associated with lung cancer risk and genome instability among never-smokers, including *XRCC1* (132), *OGG1*, *XPD* (133), and *AGT* (134). A study using nasal epithelial cells further revealed that NER was among the top 6 pathways with altered gene expression in association with third hand smoking (135). While these data underscore the potential role of environmental exposures in modulating DRC, the mechanism by which passive smoking affects the activity of specific DNA repair pathways is incompletely understood.

#### 2.3.4 Circadian Rhythm, Lifestyle, and Dietary Factors

Lifestyle factors have been shown to influence DRC. One of the most studied factors is circadian rhythm, which has been reviewed extensively (136–138) (139). It has recently been shown directly that individuals subjected to a night shift schedule exhibit diminished DRC (140). Diet can also affect the efficiency of DNA repair (141). Mounting evidence indicates that calorie restriction is associated with changes in DNA damage and repair (142). While these phenomena await more detailed study in human populations, animal models provide substantial support for the influence of diet on DNA repair. In mice, calorie restriction enhances NHEJ (143), and increases the fidelity of DNA polymerase and DNA excision repair in the liver (144). It also reverses the age-related decline in BER in brain, liver, spleen, and testis, and lowers their mutation frequency (145). In rat hepatocytes, caloric restriction altered the induction and repair of DNA damage in a manner that depended on age (146). Findings from an *Ercc1* ^Δ/-^ mouse model of premature aging further show that dietary restriction from 10% to 30% could preserve genome integrity, mitigate premature-aging associated decline in gene transcription, and prolong their lifespan (147). This supports the hypothesis that dietary restriction may attenuate the aging process. Similarly, chronic supplementation of melatonin reduces DNA damage by upregulating APE and OGG1 (148). The underlying mechanism and whether additional DNA repair pathways are affected require further investigation. Overall, the findings in humans and animal models support a role for lifestyle and circadian rhythm in DNA repair, adding a layer of complexity to the origins of inter-individual differences.

## 3 Variation in Immune Function and Its Relationship to Cancer Risk and Carcinogenesis

The immune system defends against both infection and malignancy. Based on the response time, mode of initiation, and the cell types involved, there are two immune subsystems. The innate immune system is activated rapidly upon recognition of pathogenic antigens and stress signals. It is, in part, comprised of dendritic cells (DC), monocytes, macrophages, granulocytes, and natural killer cells (NK). These cells phagocytose pathogens and activate inflammation signaling pathways and the complement cascade. The adaptive immune system, on the contrary, is more flexible in recognizing antigens. Its cellular components, including T lymphocytes (T cells) and B lymphocytes (B cells) can undergo mutagenesis to create novel and specific antigen receptors. T cells can be further subdivided into naïve T cells that recirculate between blood and lymph nodes to scout for specific antigens and memory T cells that are long lived and can mount a response to previously encountered immunogenic stimuli. Cytotoxic T cells (or CD8^+^ T cells) secrete granzymes to induce apoptosis in target cells and pore-forming perforin to punch holes in the target cell membrane for granzymatic actions. T helper cells (or CD4^+^ T helper cells) secrete cytokines to activate macrophages and further activate cytotoxic T cells. B cells express membrane-bound and secretory antigen-specific immunoglobulins (or antibodies) to defend against pathogens. Like NK cells, they are also involved in the activation of CD4^+^ T cells (149). Thus, immune response to foreign antigens depends on the specific functions of and interplay between the two immune subsystems that are comprised of a wide variety of immune cells.

Due to the presence of neoantigens that arise from genome instability and can be presented on the cell surface, cancer cells can be immunogenic. They can accordingly be recognized and eliminated by immune cells in the process of immune-surveillance (2). However, cancer cells are capable of escaping surveillance by altering antigen expression and hijacking the immune system to favor tumor growth. Through cytokine secretion, they can induce the differentiation of myeloid suppressor cells, which are inflammatory monocytes capable of inhibiting the activities of cytotoxic T and NK cells, as well as DC maturation (150). Moreover, as innate immune cells, including macrophages and neutrophils, infiltrate into tumors through chemotaxis, they can be polarized towards a pro-tumor phenotype and increase the secretion of proinflammatory cytokines to support, rather than suppress, tumor growth (151).

Current cancer immunotherapies that leverage the cytotoxicity of immune cells have proven efficacy in suppressing tumor growth. For example, NK and NKT cell populations expanded and activated *in vitro* have demonstrated potent cytotoxicity against liver cancer (152). T cells engineered with chimeric antigen receptors (CAR-T) are highly effective in targeting CD19-expressing tumors (153). DC vaccines that capitalize on the cytotoxicity of monocyte-derived DCs induce a tumor-specific immune response, although the effects differ by vaccination route and do not correlate with overall survival in phase I/II clinical trials (154). To date, immune checkpoint blockade therapies that target the cytotoxic T-lymphocyte-associated protein 4 (CTLA-4), programmed death 1 (PD-1) and its ligand PD-L1 have demonstrated improved responses and better overall survival for multiple cancers (155). Pembroluzuimab, which is an anti-PD-1 antibody, has been approved by the Food and Drug Administration to treat patients with metastatic melanoma. Another anti-PD-1 antibody, Nivolumab, has also been approved to treat patients with metastatic melanoma and patients who are previously treated for advanced or metastatic non-small cell lung cancer. These emerging therapeutic strategies form the basis for numerous ongoing clinical trials (156). For the purpose of this review, we highlight them as evidence in support of immune control of cancers.

### 3.1 Defects in Immune Function Are Linked to Cancer

Impaired immune function has been linked to increased cancer risk. By analogy to genetic diseases of DNA repair deficiency, patients with impaired immune function have provided insights on the role of the immune system in cancer. Numerous primary immunodeficiency disorders are associated with increased risk of malignancy (157) (158) (159) (160). Notably, since the DNA repair machinery plays integral roles in multiple aspects of immune function, some immunodeficiency disorders are caused by genetic defects in DNA repair as outlined in **Section 2.2**. In the general population, individuals with low cytotoxic activity of peripheral blood lymphocytes have higher risk of cancer (161). Immunosuppression due to organ transplantation and some viral infections are likewise factors that impair the immune response. Patients receiving immunosuppressants to prevent organ rejection have higher risk for non-melanoma skin cancer (162). This may explain why transplant recipients are generally more likely to develop cancer than those without organ transplantation (163–166). Cancers in transplant patients are also more aggressive and are associated with poor overall survival (167–169). Viral infection can suppress the immune system and increase cancer risk. Human immunodeficiency virus (HIV) -infected patients develop more aggressive cancer (164) and have higher risk for Kaposi’s sarcoma, B-cell non-Hodgkin’s lymphoma, and multiple myeloma (170).

Despite the strong evidence in support of a role for the immune system in controlling cancers, there are notable exceptions. Individuals with severe combined immunodeficiency due to loss of LIG4 function and those with dendritic cell deficiency tend to be susceptible to hematologic malignancies, but are not notably predisposed to solid malignancies (160, 171). Similarly, immunodeficient mice do not necessarily develop cancer. NOD scid gamma (NSG) mice have a relatively low risk of developing tumors over a life-span of about 89 weeks (172), and nude mice do not frequently develop spontaneous tumors (173), despite being highly susceptible to infection (174, 175).

### 3.2 Factors That Contribute to Variation in Immune Function

Inter-individual variation in immune function has been postulated as a driver of variations in cancer susceptibility. While age appears to be the most prominent intrinsic driving factor for variation in immune function, environmental exposures can also have a significant impact. Genetic variation associated with autoimmunity, inflammatory disease, and susceptibility to infection, is estimated to explain to 20-40% of the immune variation (176), leaving the remainder to be explained by other mechanisms. In this section, we review how immune function can be affected by heritable factors, and describe how environmental exposures may further explain inter-individual variation in the immune response across populations.

#### 3.2.1 Genetics

Reminiscent of the situation for DRC, significant inter-individual differences in immune function have been reported. In a recent detailed repeated measures study, inter-individual variation in immune cell composition and plasma cytokine levels revealed that differences between individuals are generally larger than longitudinal variability within person (177). Plasma levels of the chemokine CC chemokine ligand 20 (CCL20) are negatively associated with the proportion of central memory and effector memory cells in CD4^+^ and CD8^+^ T cell lineages, and individuals with extremely high counts of these immune subsets are found to have low levels of plasma CCL20 and CCL22. Plasma levels of IL-16 are also negatively associated with the proportion of central memory T cells in CD4^+^ and CD8^+^ lineages, and CD56^dim^ NK cells. Overall, plasma levels of 21 proteins accounted for nearly 80% of the variation in the abundance of central memory T cells. In a separate study, the abundance of CD8^+^CD45RO^+^ memory T cells and CD3^+^CD56^+^ NKT cells was found to vary significantly between individuals in repeated measures taken from 25 individuals over at least a three-week interval, but levels were largely stable within-person (178) Of note, differences in immunophenotype have been associated with age, sex, body mass index, and race (177, 179–183). Environmental exposure, vaccination (184, 185), and infection (186–189) can furthermore lead to within-person variation. Nevertheless, the observation that the immune cell composition and cytokine levels of an individual are relatively stable throughout a year (177) suggests that some variability may be determined by genetics or processes that occur during development.

Several lines of evidence support a role for genetics in human variation with respect to immune function. Although studies in monozygotic and dizygotic twins indicate that immune responses are dominated by non-heritable factors, numerous parameters including serum proteins and immune cell population composition are heritable (190). Single nucleotide polymorphisms (SNPs) in the IL-12B gene, which codes for IL-12p40, are associated with immune-related diseases such as psoriasis (191) and asthma (192). Eight SNPs have also been identified to be associated with IL-10 levels (181). Furthermore, studies in twins indicate that *ex vivo* lipopolysaccharide (LPS)-induced IL-1β production, as a measure of innate immunity, is heritable (180). This suggests that varying levels of LPS-induced secretion of tumor necrosis factor alpha (TNFα), which ranges widely from 0.187 to 2.714 ng/ml in healthy blood donors may be explained at least in part by genetics (193). In further support of genetic variation as a driver of differences in immune responses, a functional study using toll-like receptor (TLR) ligand-stimulated cord blood mononuclear cells has detailed the association between cytokine production and SNPs in innate immune genes (182). Taken together, the available data support a role for genetics in inter-individual variation in immune function in the general population.

#### 3.2.2 Aging

It has long been appreciated that the immune system undergoes age-related changes, which are collectively referred to as immunosenescence and notably include the accumulation of DNA damage in immune cells (194). Although age-dependent changes in immune cell function have been reported in bone marrow (187), bronchoalveolar lavage (179), and thymus (195), this review will focus on PBMCs because they are most immediately amenable to population studies. Several studies have found age-dependent changes in total leukocyte counts (196) or the composition of leukocyte subtypes (179, 197, 198) (199) (183, 198) (**Table 2**).

**Table 2:** Age-dependent changes in the population of immune cell subtypes.

Immune system	Cell types	Cell subtypes	Age-dependent change	Rate of change	Ref
**Adaptive immune system**	Total lymphocytes		Decrease	Not studied	(196)
	T lymphocytes	CD4^+^ T cells	Slight decrease	An average of 9.8 cells/μl/year ranging from -120 to +170 cells/μl/year	(199)
		Naïve CD4^+^ T cells (CD45RA^+^CD28^+^)	Decrease	An average of 4.3 cells/μl/year ranging from -80 to +108 cells/μl/year	(199)
			Decrease	−0.3%/year	(200)
		Treg (CD4^+^CD25^+^FOXP3^+^)	Increase	An average of 1.4 cells/μl/year ranging from -4 to +10 cells/μl/year	(199)
		CD4^+^CD28^-^ T cells	Increase	An average of 1.6 cells/μl/year ranging from -23 to +60 cells/μl/year	(199)
			Increase	0.24%/year	(200)
		CD8^+^ T cells	Decrease	An average of -1.3 cells/μl/year ranging from -163 to +69 cells/μl/year	(199)
		Naïve CD8^+^ T cells	Insignificant change	An average of -1.8 cells/μl/year ranging from -121 to +53 cells/μl/year	(199)
		CD8^+^CD28^-^ T cells	Insignificant change	An average of 0.9 cells/μl/year ranging from -121 to +53 cells/μl/year	(199)
	B lymphocytes	Mature B cells	Insignificant change	-6.6 cells/μl/year	(199)
		Naïve B cells	No difference	-5.5 cells/μl/year	(199)
			Decrease	−0.36%/year	(200)
		Memory B cells	No difference	-0.1 cells/μl/year	(199)
**Innate immune system**	NK cells		No difference	An average of 25.3 cells/μl/year ranging from -180 to 100 cells/μl/year	(199)
			Increase	Not studied	(196, 201, 202)
		CD56^bright^ NK cells	Decrease	Decrease from 15.6 cells/μl to 8.1 cells/μl in 60 years	(196)
		CD56^dim^ NK cells	Increase	Not studied	(201)
	Monocytes		Trend of increase	Not studied	(203)
	Dendritic cells	Plasmacytoid DCs	Decrease	Not studied	(203)
		Myeloid or classical DCs	Increase	Not studied	(203)

Age-related changes in adaptive immune cells have been noted. A major study involving 177 individuals, who were sampled every six months for three years, has identified an age-dependent decrease in CD4^+^ and CD8^+^ recent thymic emigrant T cells and transitional B cells (183). This decrease coincides with the reduction in thymus and bone marrow activity and an increase in the inflammatory population and CD8^+^ T cells. In particular, the proportion of CD4^+^ T cells decreases with age (198, 199) whereas that of CD4^+^ NKT cells increases with age [(198); **Table 2**]. Based on the expression of CD27 and CD28, T cells can be further subdivided into naïve and early-differentiated cells (CD27^+^CD28^+^) and fully differentiated (CD27^-^CD28^-^) CD4^+^ and CD8^+^ T cells (204, 205). A younger cohort had a significantly larger CD27^+^CD28^+^ subpopulation when compared to an older cohort (206). Similarly, based on the expression of a leukocyte common antigen isoform, CD45RA, and chemokine receptor CCR7, T cells can be subdivided into naïve (CD45RA^+^CCR7^+^), central memory (CD45RA^-^CCR7^+^), effector memory (CD45RA^-^CCR7^-^), and terminally differentiated effector memory (CD45RA^+^CCR7^-^) cells (207). Within the CD8^+^ subset, older individuals had fewer CD45RA^+^ naïve T cells and more central memory and terminally differentiated effector cells when compared to younger individuals (208). In contrast, within the CD4^+^ subset, a decrease in the CD27^+^CD28^+^ cells was the only difference observed in older individuals. These findings imply that the naïve T cells shift towards a more terminally differentiated subpopulation upon aging. This may limit the plasticity of the naïve T cells to differentiate and respond to novel antigens. The concomitant loss of the central memory and terminally differentiated CD8^+^ T subsets suggests that regardless of the activation by CD4^+^ T cells, the cytotoxic T cell response is compromised in the elderly.

Age-dependent, subset-specific changes in innate immune cell counts have been documented. The proportion of NK cells increases with age (196, 199, 201, 202) (**Table 2**) Based on the expression level of the pathogen recognition receptor CD56 (209), NK cells can be further divided into CD56^bright^, which resides in the lymph node (210) and are immunoregulators due to their cytokine production capacity (211), and CD56^dim^ NK cells, which have cytotoxic potential (212). The CD56^bright^ subset is less abundant in cord blood when compared to adult blood (202), and decreases further with age (196). By contrast, the CD56^dim^ subset increases with age (201, 203). Similarly, the proportion of monocytes increases with age (203). This monocyte population includes the classical, transitional, and CD14^+^CD16^+^ non-classical subsets. Among the non-monocytes, the proportion of myeloid-derived DCs increases with age, whereas that of plasmacytoid DCs decreases with age. In view of the age-dependent changes in immune cell composition, associating the cell count and their functions will help map the landscape of immunophenotype throughout life. Integrating this information may help identify phenotypic and functional biomarkers for immunosenescence, treatment response, or higher susceptibility to diseases including infections and cancers.

Molecular markers of immune function, including cytokine production and response to antigenic stimuli also change with age. In particular, the production of cytokines interferon gamma (IFN-γ), interleukin (IL) -4 (IL-4) and IL-6 has been shown to increase with age whereas that of IL-2, IL-10, and TNF-α decreases with age (97, 180, 183, 213). Since the cytokine production capacity of CD4^+^ T cells is invariant with age, changes in T cell subtype composition are proposed to explain age-related changes in function (206). Interestingly, expression levels of *IL-7* are lower in nonagenarians than middle aged individuals (214). Genes in the IL7R gene network are also differentially expressed between the age groups. The fact that higher *IL-7R* expression level is associated with better prospective survival suggests a role for cytokines and immune response in longevity.

The ability of T cells to respond to mitogenic stimuli is also affected by age. Aging attenuates the proliferation of PBMCs induced by stimuli including phytohemagglutinin (PHA), concanavalin A, pokeweed mitogen, and anti-CD3 (aCD3) or anti-CD28 (aCD28) monoclonal antibodies either alone or in combination (97, 197, 213). In particular, CD4^+^ T cells from elderly individuals have a lower proliferative response to staphylococcal enterotoxin B (206). Activated T cells also have lower induction of nuclear factor kappa B (NFkB) in response to anti-CD3, phorbol myristate acetate (PMA), and TNFα (215). Notably, treatment with phorbol dibutyrate and calcium ionophore A23187 induces higher nuclear translocation of NFkB in neonatal than adult T cells, though the composition of NFkB is similar between the two groups (216). Collectively, these results imply that T cells from older individuals are less sensitive to stimuli.

Similar to T cells, NK cells isolated from elderly individuals have diminished proliferation activity and CD69 induction following treatment with IL-2 when compared to the younger group (201). The response of CD8^+^ CD45RO^+^ memory T cells and CD3^+^CD56^+^ NKT cells to IL-23 also decreases with increasing age. T cell receptor repertoire diversity decreases and clonality increases with age (217). Taken together, the findings support the notion that age-dependent decrease in immune cell function, based on proliferation and cytokine production induced by antigenic stimuli, and cytotoxicity, has an impact on cancer risk. How age-dependent changes in immune function modify cancer risk warrants further investigation.

#### 3.2.3 The Environment

Exposure to environmental agents can have major effects on the immune system (218). Given that the exposure effects have been well documented, to underscore parallels with environmental agents that affect DNA repair, we will focus on how the immune system is impacted by the same cancer-causing agents (arsenic and smoking) that were discussed in **Section 2.3.3**. We will review how environmental exposure may contribute to inter-individual variation in immune function. As with DNA damaging agents, extensive experimentation has been carried out *in vitro* and with animal models to understand the biological mechanisms underlying the immune effects of environmental exposures, but they are beyond the scope of this review.

Arsenic-induced changes in the immune system are implicated by epidemiological studies. Subjects exposed to higher levels of arsenic have higher serum levels of immunoglobulin (Ig) A (219). Urinary arsenic levels are also positively associated with the number T helper (Th) 17 cells (220), whereas nail arsenic levels are associated with lower counts of CD56^+^ NK cells (221), after adjusting for confounding factors. Consistent with impairment of the immune system, lymphocytes isolated from arsenic-exposed individuals have a longer average doubling time *in vitro* (222). They also secrete lower levels of cytokines, including IL-2, IL-4, IL-6, IL-10, tumor necrosis factor alpha (TNFα), and IFNγ under basal conditions (223) and following stimulation with concanavalin A (Con A) (224). Monocyte-derived macrophages isolated from the exposed individuals display abnormal morphology, diminished adhesion, and have reduced phagocytic capacity (225). These findings indicate arsenic exposure disrupts both innate and adaptive immune responses. Notably, arsenic exposure often leads to skin lesions (219) but not necessarily cancer. Whether the immunomodulation induced by arsenic contributes to excess cancer risk in exposed populations awaits further investigation.

Early life exposure to arsenic may also impact the immune system. Children with prenatal exposure to arsenic have higher risk of respiratory illness (226), and diminished cell-mediated immune function (227). Prenatal arsenic exposure alters cord blood immune cell composition, increases the proliferation of effector T and T cells, and reduces the suppression by T regulatory (Treg) cells in a dose-dependent manner (228) (229). Prenatal arsenic exposure is also inversely associated with the percentage of naive and activated T helper memory cells in cord blood, with notable sex-dependent differences in the strength of the association (230). Moreover, lymphocytes isolated from children with prenatal arsenic exposure secrete lower levels of CX3CL and tumor necrosis factor alpha following PHA stimulation (231). Proteomic analyses of cord blood further revealed aberrant levels of chemokine (C-X-C motif) ligands, macrophage migration inhibitor factor (232), and interleukins (233). This implies that the prenatal period may be a critical window of susceptibility for disruption of immune responses by environmental arsenic exposure. Nevertheless, further studies are needed to determine whether these arsenic effects can be causally linked to higher cancer risk later in life.

Smoking suppresses the immune system (234), but the impact of passive smoking is less studied. One study involving non-smoking adult volunteers has shown that serum levels of the nicotine metabolite cotinine correlate with an increase in the naïve CD3^+^ and CD4^+^ T cell subsets and a decrease in the memory CD3^+^ and CD4^+^ T cell subsets in peripheral blood (235). Other studies have focused on immune cells in the saliva and nasal lavage, which are primary target tissues due to their proximity to the exposure route of ETS. For instance, ETS is associated with a higher percentage of phagocytic cells in the saliva (236). ETS is also correlated with the level of immunoglobulin E and immunoglobulin A in nasal lavage following exposure to ragweed (237). By contrast, ETS has no effect on the levels of cytokines IL-2, IL-5, IL-13, and IFNγ in the nasal lavage. These findings indicate that ETS has differential effects on the subsets of peripheral T cells, and may induce inflammatory responses. Interestingly, parental smoking dose-dependently decreases IFNγ production in mitogen stimulated PBMC and is associated with active wheezing in children (238). In view of the above findings, exposure to ETS in children is suggested to be associated with asthma and cancer (239). In summary, the findings reported in this section underscore the significant impacts of two exemplar environmental exposures that can modify immune function, and which are associated with increased cancer risk.

#### 2.3.4 Circadian Rhythm, Lifestyle, and Dietary Factors

As is the case with DNA repair, accumulating evidence indicates that immune function can vary substantially within an individual over the course of the day. Circulating immune cell populations undergo cyclic diurnal changes (240) (241). Among the immune cell subpopulations investigated, rhythmic changes are strongest among naïve CD4^+^ and CD8^+^ T cells (242, 243), and weakest, albeit still significant, among B cells (240). These observations have been made in both humans and mouse models (244), which have provided insights into how circadian rhythm regulates the trafficking of immune cells (245) (246). Notably, mice immunized with T cell dependent antigen trinitrophenyl-ovalbumin (OVA) in the evening have higher serum levels of antibodies when compared to those immunized in the morning (247). Consistent with these findings in animals, individuals receiving bacillus Calmette-Guerin vaccination in the morning exhibit stronger trained immunity and adaptive response when compared to those vaccinated in the evening (248). It is thus postulated that the timing of immunotherapy or cancer vaccine administration may affect the tumor suppressing effect. With these considerations in mind, chronotherapy is emerging as a novel research field that may improve the efficacy of cancer treatment (138).

Rhythmic changes in the immune cells are associated with levels of hormones and regulated by changes in cytokine levels and the expression of molecular clock genes (249–252). Levels of the stress hormone cortisol level peak near the time of awakening and then decline throughout the day (240). Its serum level negatively correlates with the abundance of circulating T cell subsets, including total CD4^+^ and CD8^+^ T cells (243). *In vitro* treatment with cortisol further shows that the suppression is most pronounced in native T cells, when compared to central memory and effector memory T cells. By contrast, the effector CD4^+^ and CD8^+^ T cells remain unaffected. Melatonin is the pineal hormone responsible for circadian synchronization (253) and its level peaks at night (240). Treatment *in vitro* with melatonin does not affect T cell proliferation upon simulation with Con A (254). However, higher salivary melatonin levels measured in the morning are associated with a higher percentage of HLA-DR^+^ monocytes and CD16^+^ lymphocytes, a higher CD4/CD8 ratio, lower lactate dehydrogenase activity in lymphocytes, and fewer CD3^+^ and CD8^+^ cells when compared to low salivary melatonin levels (255). High salivary melatonin levels in the evening are associated with a different constellation of immune system characteristics including lower phagocytic activity of granulocytes, lower CD4/CD8 ratios, and lower circulating levels of HLA-DR monocytes and CD16^+^ lymphocytes. Moreover, melatonin inhibits the secretion of T-cell independent antibodies (IgM, IgG1, IgG2b, and IgG3) in mice (247). These findings indicate that hormonal disruption of circadian rhythm can impact the immune response in complex ways.

Similar to the situation with DNA repair (139), animal models reveal that immune cells are subject to regulation by a circadian clock at a molecular level. For instance, rhythmic changes in the expression of clock genes including brain and muscle ARNT-like 1 (*Bmal1*), nuclear receptor superfamily 1, group D, member 1 (Rev-erbα) Period circadian regulator 1 (*Per1*), *Per2*, and *Clock* have been identified in mouse bone marrow derived macrophages (256), peritoneal macrophages (257), splenic macrophages, DCs, and B cells (256). In human CD4^+^ T cells, rhythmic changes in the expression of clock genes are synchronized with the production of IFNγ, IL-2, IL-4, and CD40L (258). In wild type mice, serum levels of LPS-induced cytokines display rhythmic changes (259), which are lost in *Bmal1* deficient mice. Similarly, rhythmic change in serum levels of IL-6 is lost in *Rev-erbα*−/− mice (259). These findings reveal that the rhythmic control of immune function is tightly regulated by an intrinsic circadian clock, and the available data currently support a stronger role of the circadian clock in the innate immune response.

How nutrition modifies the immune system is a continually evolving field of research. Early studies focused primarily on the effects of vitamins and trace elements on the immune function have been reviewed (260). For instance, deficiency in vitamin B6 impairs lymphocyte maturation, proliferation, antibody production, and activity of T cells. It also attenuates NK cell activities. Deficiency in folate attenuates proliferation of CD8^+^ T cells and NK cell activities. Deficiency in vitamin B12 reduces total lymphocyte counts and the number of CD8+ cells. Vitamin C has also been shown to stimulate neutrophil chemotaxis, but its anti-inflammatory effects remain incompletely understood. Deficiency in vitamin A impairs phagocytosis and increases production of IL-12 and TNFα, which promotes inflammation. Deficiency in vitamin D impairs the innate immune response. Deficiency in trace elements including selenium, zinc, copper, and iron, can also disrupt the immune system. Comparisons between high and low fat diets have revealed impacts on cytokine levels that may impact the homeostatic balance between Treg and Th17 cells (261). Children following a Mediterranean diet for a year have higher salivary levels of an anti-inflammatory cytokine IL-10 and lower levels of IL-17 (262). A variety of dietary components, including red grape polyphenols, prebiotics, probiotics and symbiotics have been suggested to boost immune function in older individuals (263). Taken together, these findings establish an important role for diet-dependent immune-modulation, which may affect cancer susceptibility, as has been recently reviewed (263–265).

Several lines of evidence support a role for exercise in modulating immune function. Regular exercise and physical fitness can delay the onset of immunosenescence and tumorigenesis (265). Exercise improves the circulation and function of innate immune cells (266–268). Although the increase in immune cells is transient, it leads to a 40-50% decrease in the number of days with upper respiratory tract infection among adults during winter season (269). By contrast, exercise routines that induce muscle and tissue injury are pro-inflammatory and suppresses immune response transiently (265). Thus, the effects of exercise on the immune system appear complex and require further investigation.

Collectively, the data presented in this section outline numerous potential non-heritable sources of inter-individual variation in immune function. Taken together with the effects of aging, genetics, and the environment, these findings are consistent with a highly dynamic model of immune function. As with DNA repair, assessments of immune function at the individual level may provide important insights into disease susceptibility, but must be carried out in a manner that takes the many sources of variability into account. In the next section, we discuss a possible strategy for surveying both immunophenotype and genome integrity in human populations.

## 4 Potential for Simultaneous Profiling of Immunophenotype and Genome Integrity for More Accurate Assessments of Cancer Risk

**Figure 2:**
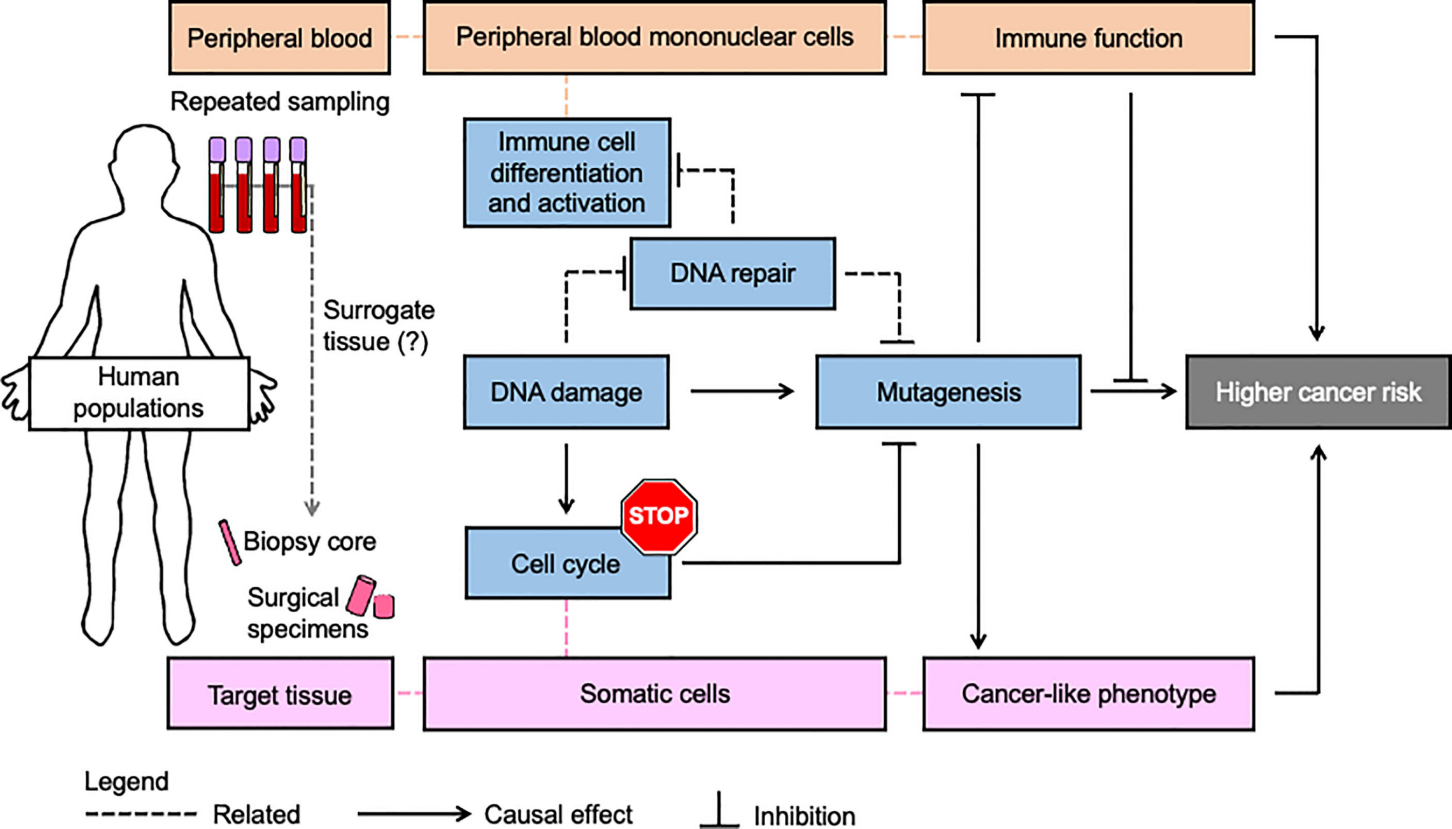
Simultaneous assessment of genome integrity and immune function may be a more robust strategy for personalized prevention and treatment of cancer. Most population studies use blood samples to assess genome integrity and immune function because blood draws are less invasive than the procedures for collecting other tissues from human subjects. A key assumption is that fundamental processes in cancer etiology (blue boxes) as measured in blood (red boxes) are sufficiently related to be considered a surrogate for the corresponding target tissue (pink boxes). Since blood and its components are heavily involved in immune processes, this tissue can provide extensive insights into immunophenotype. Likewise, lymphocytes provide extensive insights into inter-individual variation in genome integrity mechanisms, including those underlying risk of numerous solid malignancies as reviewed herein. In addition to its role in preventing mutagenesis and immunosuppression that can be induced by DNA damage, DNA repair is extensively involved in the differentiation and activation of immune cells. Nevertheless, variation in immune function and genome integrity pathways is independent and challenging to predict from genetics and indirect genomic markers. Therefore simultaneous functional assessment of DNA repair activities and immune function in studies using blood may improve the accuracy and precision of cancer risk estimates beyond what is possible when considering either process alone.

Although DNA repair and immune function are distinct biological processes, they are subject to many of the same influences, and they both play important roles in cancer susceptibility. It has long been appreciated that several DNA repair pathways play integral roles in the immune system (270) (271). Furthermore, one of the most acute consequences of exposure to DNA damaging agents is suppression of the immune system (272) (273) (273). As outlined in this review, environmental exposures such as arsenic and passive smoking, circadian disruption, and lifestyle factors can modulate both DNA repair and immune function. It is noteworthy that defects in DNA repair and immune function are two of the most prominent hallmarks of cancer (2). Accordingly, efforts are underway to perform functional profiling in human populations, with the goal of identifying biomarkers that could be used for personalized prevention and treatment of cancer. While the idea of functional profiling has been framed independently in the context of genome integrity (13), and immune function (274), we propose that considering both simultaneously would increase the accuracy and robustness of cancer susceptibility predictions (**Figure 2**).

Patients with defects in nucleotide excision repair provide an excellent example of elevated cancer risk in individuals who are deficient in DNA repair and, perhaps, immune function (275–277). XP patients have a massively higher risk of developing UV- induced skin cancers (278), but also have an increased risk of developing internal tumors including glioblastoma, leukemia, lymphoma, and lung cancer (279, 280). The prevailing hypothesis regarding cancer susceptibility, both in XP patients and among those with lower NER capacity in the general population (14), has been that increased genome instability leads to higher rates of mutation and thus greater cancer susceptibility. However, it was noted in early case reports and small studies that XP patients also suffer from some forms of immune dysfunction (281) (275). Lymphocytes from XP patients have a larger clone size in response to allogeneic leukocytes (282), suggesting that lymphocytes of XP patients are more diverse, possibly due to a higher somatic mutational burden. Earlier studies have shown that lymphocytes from XP patients are less responsive to stimulation with mitogens (275, 277). Notably, serum from XP patients can attenuate the response of normal lymphocytes to PHA (275). A case study has also shown that DCs isolated from a trichothiodystrophy (TTD) patient with an XPD mutation have lower expression of CD86 co-stimulatory molecules and HLA glycoproteins, and are defective in stimulating native T lymphocytes (277). Notably, TTD patients commonly suffer from infections and there are several documented cases of immunodeficiency (283). Since some TTD patients do not appear to exhibit defects in DNA repair, these findings raise the possibility that NER proteins could have a role in immune function that is distinct from their role in DNA repair. NK cells from XP patients of multiple complementation groups display impaired lytic activity and lower IFNγ production in response to poly I:C stimulation, though the total NK cell count is normal (276). Moreover, XP patients have higher tolerance to the grafting of skin from a normal HLA-incompatible donor (275). Taken together, these findings suggest that innate and adaptive immune cell function may be defective in patients with nucleotide excision repair defects, but the underlying mechanism and the extent to which these findings may extend generally to patients with XP and other NER deficiency disorders remain unknown. Additional, comprehensive studies in larger cohorts of patients with NER deficiency are needed to assess whether their cancer-prone phenotypes can be explained in part by an accompanying immune defect. Such studies would also illuminate whether inter-individual variation in NER can be expected contribute to variation in immune function in the general population.

In the case of xeroderma pigmentosum variant (XP-V), patients express a truncated *POLH*, which reduces the expression and activity of DNA polymerase η (Pol η) (284). POLH is involved in translesion synthesis (TLS), which promotes tolerance of CPDs, thymidine dimers, and 8-oxoguanine lesions (285, 286). Loss of *POLH* leads to error prone-repair of CPDs by mutagenic polymerases zeta, kappa, and iota (287). Interestingly, UVA irradiation induces a mutational signature that suggests a role for basal mutagenesis induced by oxidative damage in the elevated risk for internal cancers in XPV patients (288). XP-V patients also have lower frequency of A/T mutation and higher frequency of deletion in Ig genes in activated B cells, which likely reflects the role of POLH in somatic hypermutation in B cells (289). POLH deficiency may thus drive the higher cancer incidence among XP-V patients *via* multiple mechanisms. In contrast with the severe combined immunodeficiency often associated with LIG4 syndrome due to disrupted V(D)J recombination (35), XPV patients do not present with pronounced immunodeficiency, possibly due to compensatory activities of other polymerases in somatic hypermutation. A small group of patients deficient for a subunit of another polymerase (POLE) does exhibit immunodeficiency and points to the possibility for additional rare polymerase deficiency disorders yet to be discovered (290).

Population studies offer numerous opportunities for simultaneous investigation of immune function and genome integrity. In identifying cancer risks and associating genome instability with cancer outcomes, these studies almost exclusively rely on blood samples due to its safe and relatively less invasive sampling method when compared with other types of biopsies. Furthermore, the multitude of cellular and molecular markers of immune function in blood represent a rich source of information that can be paired with analyses of genome integrity in lymphocytes. Some studies have already taken advantage of the opportunity to measure both genome integrity and immune function in a single population. For example, it has been observed that immunosuppressive drugs suppress DNA repair in human PBMCs (291, 292). As discussed in the following section, emerging technologies have greatly increased the feasibility of simultaneous profiling of DRC and immunophenotype in human populations.

## 5 Technological Advancements That Will Help Shape the Future of Precision Medicine

Significant technological advances have recently yielded functional tools for the interrogation of genome integrity and immune function. Here we review a sampling of emerging technologies that hold promise for enabling combined phenotyping with respect to DNA repair and the immune system in human populations.

As has been reviewed recently, several technologies are now available for analyses of genome integrity in human populations (13). Fluorescence-based multiplex flow-cytometric host cell reactivation (FM-HCR) assay measures the ability of live cells to repair site-specific DNA lesions (293). The assay is designed to have each fluorescent plasmid engineered to incorporate a specific type of DNA damage, including mismatches, abasic sites, oxidized bases, or DSB. The use of multiple fluorescent proteins enables multiplexing analyses for DNA repair activities. FM-HCR has thus been applied in a variety of settings, including in primary human lymphocytes (294–299).

The high throughput CometChip has been developed based on the established single gel electrophoresis assay (300, 301). Due to its 96-well format and automated image analysis, the CometChip is amenable to analyse large numbers of samples. It has recently been applied in a population study (302) and has been widely adopted for genotoxicity testing (303–305). CometChip technology has also been modified to interrogate DNA methylation status (306), levels of specific DNA adducts (307), and DNA damage in spheroids, which is also known as SpheroidChip (308).

A fluorescence-based unscheduled DNA synthesis (UDS) assay provides a substantially more convenient and user-friendly approach for measuring NER in populations. The original UDS assays used radio-labeled thymidine and autoradiography, making them laborious and inconvenient for routine clinical use (309). A new fluorescence-based method incorporates a thymidine analogue 5-ethynyl-2-deoxyuridine, which is conjugated to a fluorescent azide after UV irradiation and can be quantified by flow cytometry (310) (311). This technology is now being used to support the diagnosis of rare DNA repair deficiency disorders (312).

Single-cell whole-genome sequencing has opened up a new venue for studying somatic mutation and identifying mutational hotspots within the genome (313–316). This technology leverages single-cell multiple displacement amplification (SCMDA) procedure for detecting a full spectrum of base substitutions in a somatic cell. The technology has been used to reveal age-dependent changes in somatic mutations of B lymphocytes. The mutations in normal B lymphocytes not only resemble the COSMIC signatures in cancer (317), the data imply the age-dependent accumulation of somatic mutation is pivotal to the development B cell cancers (316). Thus, SCMDA, in combination with single-cell whole genome sequencing, is the tool for dissecting interindividual variation in mutation burdens influenced by genetics, age, environmental exposure, and lifestyle factors.

Single-cell RNA and DNA sequencing technology has advanced rapidly in recent years and found application in nearly every dimension of human biology (318). This technology analyzes the transcriptome of single cells within a heterogeneous population (319). It provides a powerful unbiased alternative to immunophenotyping approaches such as flow cytometry mass cytometry (CyTOF), which are less expensive but require labeling of surface markers and reveal little additional information at the single cell level (320) (321) (322). Single cell RNA sequencing enables the interrogation of cell-cell interactions, identification of changes during cell fate specification, and dissection of regulatory networks associated with cellular functions at single-cell level and based on cellular subtypes, which are not feasible in whole tissue analyses (323–326). Although single cell technologies remain expensive, continuous innovation raises the prospect of their eventual application in population studies. The emerging theory of clonal hematopoiesis of indeterminate potential (CHIP) describes the presence of somatic mutation in the cancer driver gene at a variant allele frequency of at least 2% in blood and bone marrow cells of a healthy individual (327–329). This process of clonal selection effectively amplifies mutations in a manner that makes them detectable by bulk sequencing. CHIP is induced by DNA damaging agents, and associated with increased risk of both leukemia and solid malignancies. It can thus be presumed to represent a molecular ruler that reflects both exposure to DNA damaging agents and the ability to repair DNA damage at the individual level.

Cellular indexing of transcriptomes and epitopes by sequencing, also known as CITE-seq, is a high throughput single-cell RNA sequencing analysis that is coupled with epitopes to interrogate expression of cell surface proteins (330). Since immune cell subtypes express specific surface markers, which can be captured by specific epitopes, CITE-seq has been widely used for determining the transcriptome profile of specific immune cells within a heterogeneous population (331, 332). Though CITE-seq and single-cell RNA sequencing serve similar purposes, CITE-seq has a shallower sequencing depth and relies heavily on the protein expression of specific cell surface marker. Its design better fits for studying immune cells.

Historically, it has not been feasible to perform functional screens of such nuanced phenotypes as those associated with modest defects in genome integrity or immune function. But these emerging technologies, particularly when used in combination, will enable such studies. Since blood samples are routinely collected for molecular epidemiological studies that focus on either genome integrity or immunophenotyping, the tissue could be maximally leveraged to understand how both processes may interact and contribute to cancer risk. Furthermore, studies combining immunophenotyping with genome integrity assays may shed light on whether mild DNA repair deficiencies in the general population lead to increased cancer risk, at least in part, by limiting the efficiency of immune responses.

## 6 Open Questions and Future Strategies for Population Studies

Here we briefly propose a framework for future studies aimed at understanding the joint influence of inter-individual variation in DRC and immune function on cancer risk. We pose several questions in the field that we view as important areas to investigate, followed by broad recommendations for pursuing population studies at the intersection of immune function and genome integrity.

### 6.1 Open Questions

**Figure 3:**
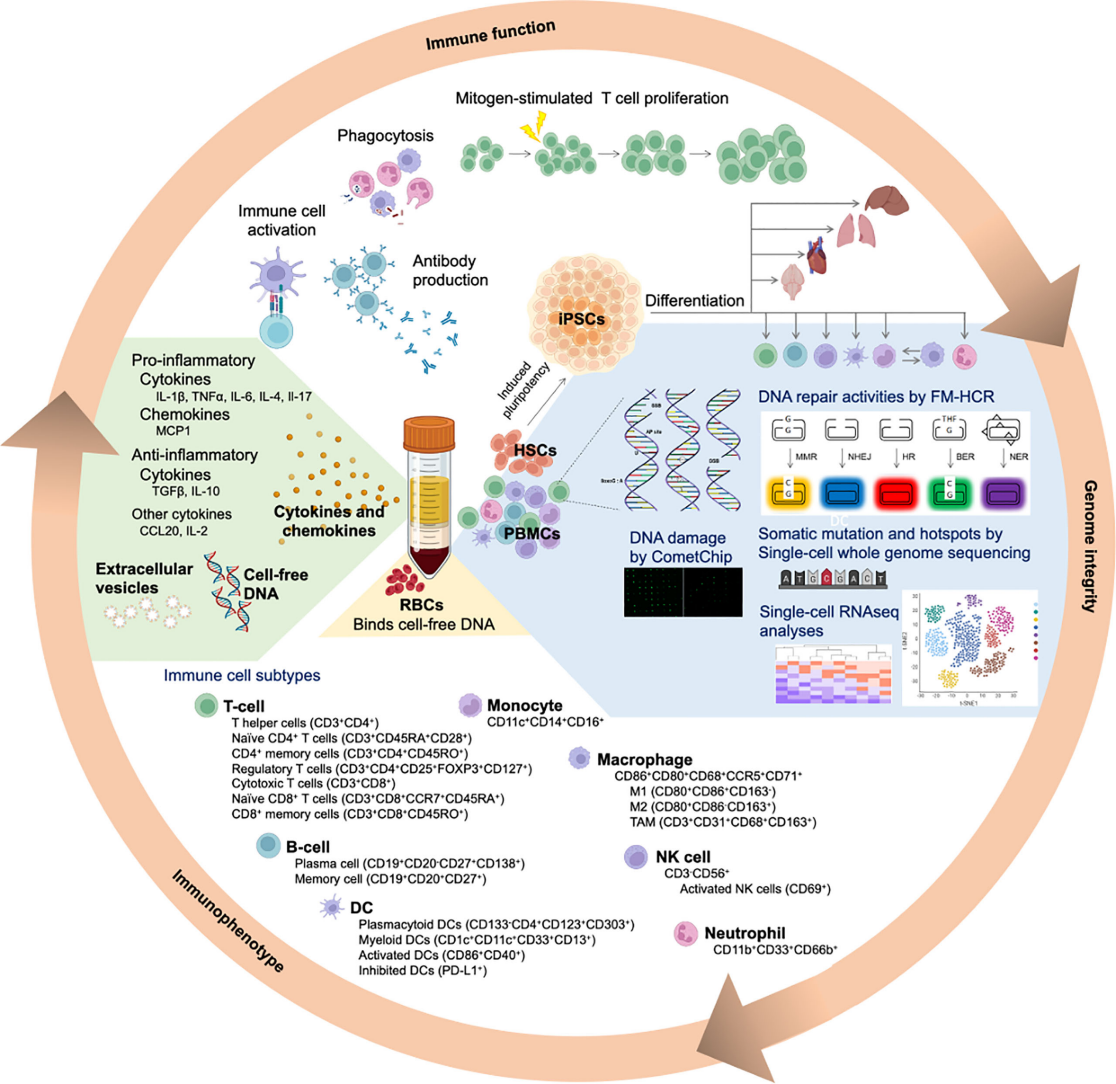
Simultaneous assessment of genome integrity and immune function using human blood samples. Following density gradient centrifugation of peripheral blood, peripheral blood mononuclear cells (PBMCs) are enriched in the buffy coat layer. Different immune cell subtypes within the PBMC population can be further identified based on their specific cell markers. Genomic integrity of the immune cell subtypes can be comprehensively evaluated by integrating various complementary approaches. Fluorescence-based multiplex host cell reactivation (FM-HCR) evaluates the ability of cells to repair specific DNA lesions. The CometChip assay reveals the magnitude of genomic DNA damage and repair kinetics in a high throughput manner. Single-cell whole genome sequencing identifies somatic mutations, whereas RNAseq (CITE-seq and single-cell RNAseq) measure the transcriptome. Moreover, hematopoietic stem cells isolated from the blood sample can potentially be used to generate induced pluripotent stem cells (iPSCs). Upon differentiating these iPSCs into a somatic cell type of interest, it becomes feasible to obtain large number of patient-derived, tissue-specific somatic cells, which may otherwise be scarce or not feasible to obtain. Red blood cells (RBCs), which are enriched in the bottom layer, bind cell-free DNA to minimize inflammatory responses. The plasma layer contains cytokines and chemokines secreted from the immune cells. These signaling molecules can be pro-inflammatory or anti-inflammatory, depending on the cellular status and presence of antigens. Notably, cell-free DNA and extracellular vesicles (EVs) are present in the plasma.

1. **Is blood a reliable surrogate for other tissues?** Blood is an extremely rich source of data, including a variety of immune cells, cytokines, circulating DNA, and small molecules that can be analyzed to assess immune function, DNA damage and repair, and environmental exposures (**Figure 3**). Because it can be collected relatively easily and in a repeated manner, sampling blood is also among the most feasible approaches for population studies. Nevertheless, circulating immune cells may not reflect the biology of tissue resident immune cells and tissue-specific microenvironments. For these reasons, whenever possible, ideal studies would include sampling the tissue of interest and, in the case of cancer studies, the tumor as well.

2. **Which immune markers are the best predictors of cancer risk and outcomes?** The emerging technologies described in the previous section provide an unprecedented opportunity for deep analysis of immunophenotypes, but because they have been developed so recently, they have only begun to be applied towards understanding the relationship between immune function and carcinogenesis. Studies surveying a broad array of immune markers are needed; these would include a census of circulating immune cells, measurements of cytokines, and tests for immune cell function.

3. **Which combinations of functional assays are the best predictors of cancer risk and outcomes?** Emerging functional assays described above and numerous established assays for immune cell activation and proliferation (333) integrate complex regulatory mechanisms and can complement ‘omics approaches (genotyping, transcriptional profiling, proteomics and DNA sequencing). Functional assays for DNA repair often outperform polygenetic cancer risk scores (334), and even stronger associations are seen in limited cases where multiple functional assays for different pathways have been applied to the same set of samples (335). But it is not possible to predict which functional biomarkers provide the most useful information to support mathematical models that would predict cancer risk or cancer outcomes. Thus, cancer case-control studies should be designed to integrate as many functional assays as is feasible for the same set of subjects. Given the practical constraints of funding and expertise, biological materials should be banked appropriately to enable future analyses.

4. **How do markers of genome integrity and immune function change over the life course?** As detailed in **section 2** and **section 3**, the phenotypic markers we propose to survey with the goal of advancing personalized medicine are subject to time-dependent variation due to a variety of factors including lifestyle, environmental exposures, health status, and aging. To use these functional biomarkers as predictive tools, it will be necessary to carry out longitudinal studies wherein they are measured prospectively.

5. **Does NER contribute to immune function?** Numerous DNA repair pathways are already implicated in the mutagenic processes that occur during immune cell development and activation. In addition to those processes, emerging roles for DNA damage and DNA repair in gene regulation (336, 337), together with the growing recognition that many proteins “moonlight” in multiple roles within the cell (338), raise the possibility of as yet unrecognized mechanisms by which DNA repair pathway might contribute to immune function. By carrying out detailed immunophenotyping in individuals with profound defects in DNA repair, such as patients with XP, CS, and TTD, it can be determined whether NER deficiency, perhaps specifically which global genome (GG-NER) or transcription-coupled (TC-NER) NER subpathways, is associated with an immune disorder.

6. **Can stem cell-derived cells recapitulate DRC of primary human tissues?** A growing number of studies have found associations between DRC in blood cells and cancer risk, and the simplest interpretation is that the blood cells accurately represent genome maintenance in the tissue where the cancer develops. However, DNA repair varies with cell type and as a function of cell cycle and the tissue microenvironment. It is therefore possible that at least some of the associations between cancer risk and genome integrity as measured in immune cells is a reflection of immune cell function, rather than genome integrity in the target tissue. This question can in principle be unraveled by studies that measure DNA repair in multiple cell types from the same individual, but it likely will not be feasible to collect most tissues as part of a population study. By differentiating stem cells into cell types of interest, it may be possible to recapitulate physiological cell programming and make tissue-specific assessments of DRC on an individualized basis.

### 6.2 Recommendations

1. **Focus on human studies:** The framework we are proposing is at least in part discovery-based and centers human subjects, not biological model systems. This is a notable departure from the traditional approach more familiar to mechanistic biologists, wherein simple genetic models are used to test hypotheses before broaching the complexity of human systems. Instead, in this framework, one would first identify promising biomarkers in humans, and then follow up with confirmatory studies in model systems that best approximate the human biology. Taking the differences in telomere biology in mice and humans as an example (339), one can appreciate the value of prioritizing mechanistic characterization of biomarkers that have shown promise in human studies, and doing so in a model system that recapitulates the human biology. Though highly controlled genetic model systems such as CRISPR knockouts are not a feature of population studies, there are invaluable natural experiments and edge cases that can be leveraged for analogous purposes. For example, the phenotypes associated with rare genetic disorders that disrupt key aspects of genome maintenance and/or immune function such as those discussed in previous sections can be taken as upper or lower bounds for phenotypic variation in the general population. Likewise, biological samples from patients undergoing therapy with immunogens, immunosuppressants, or DNA damaging agents provide opportunities to understand physiological human responses to potentially carcinogenic real-world exposures. This is particularly so when the studies are conducted longitudinally, such that functional assays can be applied to samples collected before and after the exposure. Samples from individuals participating in studies that collect detailed personal environmental monitoring data present similar opportunities, and hold the advantage of avoiding the potential bias introduced by focusing on individuals with pre-existing health conditions, as is common in clinical studies.

2. **Maximize the use and preservation of biological sample(s):** The comprehensive functional characterization of human populations we are proposing is ambitious and may require some realignment of funding agency priorities and philosophies to reach its full potential. The prioritization of hypothesis-driven research commonly constrains the scope of projects and forces researchers to make decisions to severely limit the collection and analysis of biological samples. However, as illustrated in **Figure 3**, biological samples have extraordinary potential to provide insights into the many mechanisms driving human variation. To address this mismatch in the meantime, researchers should preserve biospecimens as comprehensively as possible. In the case of blood samples, this would entail banking each of the components and preserving them in a manner that is compatible with future downstream analyses, which may require live cells, for example.

3. **Engage in team science:** Population studies that make use of emerging technologies to characterize biological samples are inherently interdisciplinary. It is generally not within the capacity of a single investigator to have the expertise needed for establishing a human study cohort, developing and applying new technologies, interpreting biological data that span multiple fields, and, when applicable, treating and evaluating patients. In addition to a diverse group of scientific and medical experts who cover the technical expertise, the team should ideally include stakeholders who stand to benefit from the research. These stakeholders can also guide the focus of the study from its inception and ensure that vulnerable and underserved populations are included.

## 7 Closing Remarks

Many of the syndromes associated with defects in immune function or genome integrity have been discovered in recent years as genotyping technology has advanced. But these studies importantly relied upon functional characterization of variants of unknown significance, or the discovery of patients with a familiar disease of unknown etiology. The data suggest there are many more deficiency syndromes still to be discovered. Functional assays such as those outlined herein present powerful tools for identifying individuals with deficiencies in immunity or genome maintenance. By integrating these assays with modern genomics tools, it should be possible to accelerate the discovery and annotation of rare variants as well as functional associations with disease. Population studies are most easily carried out with blood, which contains the circulating cells and cytokines that can be used to define the immunophenotype. Therefore blood samples represent a largely untapped resource for analyzing both genome integrity and immune function simultaneously. Studies that compare these biological features between cancer patients and healthy counterparts will provide important clinical insights. Yet, simply surveying the complexity of the functional landscape across populations to define the range of variability is also a useful precursor to developing predictive models that incorporates the variability to explain disease susceptibility. Leveraging the advanced technologies and our current understanding of DRC, immune function, mutation, and cancer, it is timely to address these questions and improve the precision of strategies that assess and manage cancer risk for the welfare of population health.

## Author Contributions

Both AC and ZN developed the concept and defined the scope of the review. AC performed a literature review, organized the results, drafted the review, and composed the figures and tables. ZN supervised the development of the review and contributed to the writing and editing of the manuscript and its components.

## Funding

This work was supported by awards U01ES029520 and P30ES000002 (PI Weisskopf) to ZN. Both awards supported salary for ZN. An internal (Harvard T.H. Chan School of Public Health) award to ZN provided salary support for AC.

## Conflict of Interest

The authors declare that the research was conducted in the absence of any commercial or financial relationships that could be construed as a potential conflict of interest.

## Publisher’s Note

All claims expressed in this article are solely those of the authors and do not necessarily represent those of their affiliated organizations, or those of the publisher, the editors and the reviewers. Any product that may be evaluated in this article, or claim that may be made by its manufacturer, is not guaranteed or endorsed by the publisher.
